# IGF1 genotype, mean plasma level and breast cancer risk in the Hawaii/Los Angeles multiethnic cohort

**DOI:** 10.1038/sj.bjc.6600728

**Published:** 2003-01-28

**Authors:** K DeLellis, S Ingles, L Kolonel, R McKean-Cowdin, B Henderson, F Stanczyk, N M Probst-Hensch

**Affiliations:** University of Southern California, Norris Comprehensive Cancer Center, 1441 Eastlake Ave., MS 44, Los Angeles CA 90033-0800, USA; Cancer Etiology Program, Cancer Research Center of Hawaii, University of Hawaii, Honolulu HI 96813, USA; USC Keck School of Medicine, Obstetrics/Gynecology, 1M2 Women's and Children's Hospital, Los Angeles CA 90089-9032, USA; University Hospital Zürich, Cancer Registry Zürich, F SON 6, Sonneggstr. 6, 8091 Zürich, Switzerland

**Keywords:** IGF1, insulin-like growth factor-1, breast cancer, ethnicity

## Abstract

The insulin-like growth factor 1 gene (*IGF1*) is a strong candidate gene for a breast cancer susceptibility model. We investigated a dinucleotide repeat 969 bp upstream from the transcription start site of the *IGF1* gene for possible associations with plasma IGF1 levels and breast cancer risk in a multiethnic group of postmenopausal women. Furthermore, we investigated the relation between race/ethnicity, mean plasma IGF1 levels and breast cancer rates in the Hawaii/Los Angeles Multiethnic Cohort. The mean age-adjusted IGF1 level among Latino-American women, 116 ng ml^−1^, was statistically significantly lower than the mean age-adjusted IGF1 levels for each of the three other racial/ethnic groups, African-American, Japanese-American and Non-Latino White women (146, 144 and 145 ng ml^−1^, respectively) (*P<*0.0001). Latino-American women have the lowest breast cancer rates of any racial/ethnic group in the cohort. These results support the investigation of an expansion of the hypothesis for an important role of IGF1 in breast cancer tumorigenesis to different racial/ethnic groups and to postmenopausal women. It is unlikely that any involvement of IGF1 in breast cancer aetiology is mediated by the *IGF1* dinucleotide repeat polymorphism, which was not significantly associated with circulating IGF1 levels nor breast cancer risk in this study. Research into relevant determinants of IGF1 levels in the blood must continue.

The insulin-like growth factor 1 (IGF1) protein has been implicated in breast cancer because of its mitogenic and antiapoptotic effect on mammary epithelial cells (Furlanetto and DiCarlo, 1984; Westley and May, 1994; Kleinberg, 1998). Epidemiological work epitomised by a paper from Hankinson *et al*, (1998) has shown an increased risk for breast cancer in premenopausal women with high prediagnostic plasma IGF1. Byrne *et al*, (2000) subsequently showed that mammographic density, one of the strongest breast cancer risk factors, was positively correlated with plasma IGF1 in premenopausal but not postmenopausal control women. Although these initial findings were limited to premenopausal women, postmenopausal IGF1 levels may also be an important determinant of breast cancer risk if considered as a component of lifetime or intratissue exposure. Assessment of lifetime or intratissue exposure may be improved by the availability of genetic determinants. An early report suggested that the homozygous status for a (CA)_19_ microsatellite variant 969 bp upstream from the transcription start site in the IGF1 gene (*IGF1*) was predictive of low serum IGF1 in Caucasian men and postmenopausal women. We hypothesised that the (CA)_19_ homozygous genotype (19/19) might indicate women with a decreased lifetime exposure to IGF1 and consequently, a decreased susceptibility to breast cancer. We examined the role of IGF1 in postmenopausal breast cancer among women from four major racial/ethnic groups (African American, Japanese American, Latino American, White) in the Hawaii and Los Angeles Multiethnic Cohort. We first examined whether the mean plasma IGF1 levels were correlated with patterns of breast cancer risk by the racial/ethnic group. We then attempted to confirm whether a microsatellite variant for *IGF1* was predictive of plasma IGF1 levels in different racial/ethnic groups and to test whether the variant was a marker of breast cancer risk in our postmenopausal multiethnic population.

## Materials and methods

### Study subjects

Participants included in these analyses were selected from a large multiethnic cohort (MEC) study in Hawaii and Los Angeles initiated with emphasis on diet and lifestyle characteristics in the aetiology of cancer. The cohort was established between 1993 and 1996 from driver's licence files in Hawaii and Los Angeles, including 215251 men and women, ages 45–75 years at the time of enrollment. The MEC includes primarily African Americans, Japanese Americans, Native Hawaiians, Latino-Americans and Non-Latino Whites. Baseline data were collected on all cohort members via a mailed questionnaire that contained five sections: (a) background, including medical history and family cancer history; (b) diet history; (c) medication use; (d) physical activity and (e) female reproductive history, including the use of hormones. Details of the study have been published previously (Kolonel *et al*, 2000). For setting up nested case–control studies, breast cancer cases and potential controls were recontacted by letter and phone call, followed by a home visit to collect a blood specimen. Blood draw was completed in the morning, typically at the person′s home, after informed consent was obtained. Participation rates for providing a blood sample on request were 74% for cancer cases and 66% for cohort controls. Case ascertainment was completed through the Surveillance, Epidemiology and End Results (SEER) cancer registries in Hawaii and Los Angeles.

We tested the association between circulating plasma IGF1 levels and racial/ethnic group in 406 healthy, postmenopausal women. For a subset of these women (230 randomly selected from the four racial/ethnic strata), genotyping of the IGF1 (CA)_*n*_ microsatellite variant also was completed for the assessment of phenotype/genotype correlation. We selected postmenopausal women with no history of cancer, who had not taken hormone therapy within 2 weeks of blood draw. A subject was defined as postmenopausal, if she met any one of three criteria: (1) over age 55 and periods stopped; (2) 55 years or younger, periods stopped and no hysterectomy; (3) 55 years old or younger, periods stopped and bilateral oophorectomy. At the time the sample was selected (January, 2000), plasma was available for IGF1 testing among 123 African-American, 71 Japanese-American, 154 Latino-American and 58 Non-Latino White postmenopausal women.

A total of 800 postmenopausal women (400 cases and 400 controls) from the same four racial/ethnic groups were selected for a nested case–control study of the microsatellite (CA)_19_ variant and breast cancer risk. Women included in the case–control analysis were selected irrespective of hormone therapy status. Of the 800 women, 56 (29 cases and 27 controls) reported having a prevalent breast and/or uterine cancer on their questionnaire and were subsequently excluded from the analysis. Three women were missing weight or height values. The remaining 368 cases and 373 controls were successfully genotyped. A subset (134/373; 36%) of the controls selected for this case–control data set overlapped with controls selected for the plasma IGF1 by racial/ethnic group analysis described above.

### IGF1 plasma levels

Plasma IGF1 was quantified in the Reproductive Endocrine Research Laboratory at USC, under the supervision of Dr Frank Stanczyk. IGF1 was measured by direct radioimmunoassay (RIA) using commercial kits obtained from Quest Diagnostics at the Nichols Institute (San Juan Capistrano, CA, USA). All samples were analysed in a single batch, meaning that kits with identical lot numbers were used and all assays were performed by the same individual. The intra-assay coefficients of variation for the RIA assay ranged from 9.5 to 13.8% (Carmina *et al*, 1995, 1999).

### IGF1 (CA)*_n_* genotyping

Genotyping of the microsatellite (CA)_*n*_ variant was performed using PAGE gel electrophoresis. We performed PCR amplification of the DNA region surrounding the microsatellite repeat in question using identical primers to those used in the study by Rosen *et al*, (1998). Their sequences were as follows 5′GCTAGCCAGCTGGTGGTGTTATT3′ and 3′ACCACTCTGGGAGAAGGGTA5′. All PCR was performed using a PTC-100 Thermocycler (MJ Research, Waltham, MA, USA). We extracted DNA from the buffy coats of peripheral blood samples using the Puregene genomic DNA isolation kit (Gentra Systems, Minneapolis, MN, USA). We used a modified genotyping protocol as described by Rosen *et al*, (1998). A total of 20 ng of DNA template, 1.25 pmols of each primer, 0.25 *μ*M of each deoxynucleotide triphosphate, 2.5 *μ*M MgCl_2_, 2% dimethyl sulphoxide, 1.5 U Taq polymerase (Promega, Madison, WI, USA) and the manufacturer's recommended buffers were combined in 25 *μ*l reactions. The forward primer was labelled with ^33^P using T_4_ polynucleotide kinase (Amersham-Pharmacia, Piscataway, NJ, USA). A ‘touchdown’ PCR cycling protocol was used which consisted of 35 cycles in total. The programme started with a 45 s denaturation at a temperature of 94°C. The first cycle continued with a 30 s annealing phase at 64°C and finished with a 30 s 72°C extension. The annealing temperature was decreased by 1°C in each of the next nine cycles, and then was maintained at 55°C for 25 cycles. Denaturation at 94°C for 45 s, and extension at 72°C for 30 s were consistent throughout the entire programme. The final extension was held for 5 min at 72°C. The radiolabelled, denatured PCR product was screened on a polyacrylamide gel by electrophoresis. Autoradiographs were exposed for 12–18 h. Two investigators scored all genotypes independently and random samples were rerun periodically to check consistency across the entire sample. The same (CA)_19_ homozygote control and a (CA)_21_ homozygote control were run on each genotyping gel. The noninformative samples were repeated in subsequent gels thereby reducing our noninformative rate to less than 1% among cases and controls.

In order to orient the genotype information attained from the PAGE gel electrophoresis assay in terms of the number of dinucleotide repeats, we sequenced a number of homozygous samples using an ABI 3700 automatic sequencer (ABI, Foster City, CA, USA). Two independent investigators read the sequencing output and were able to identify which sample contained the (CA)_19_ homozygote, thus allowing us to orient the other genotypes in relation to the (CA)_19_ on the PAGE gels.

### Data analysis

Analysis of variance (ANOVA) was used to test differences in crude and age-adjusted mean IGF1 by racial/ethnic group and *IGF1* genotype. We used a modified categorisation scheme for the *IGF1* (CA)_*n*_ genotype based on the paper by Rosen *et al*, (1998) (non-19/non-19, non-19/19 and 19/19), given their prior finding of an association between the (CA)_19_ genotype and IGF1 concentration in the blood. Means presented are least-squares means. The square root transformed plasma IGF1 levels produced the best approximate normal distribution, but the results of an analysis of the data using square-root transformations did not differ from results using data that had not been transformed. We therefore present the straightforward means derived from the nontransformed plasma IGF1 values. Odds ratios for association between the genotype and breast cancer risk were calculated using unconditional logistic regression. Odds ratios were calculated for the *IGF1* genotype categorised by the number of 19 alleles (CA_19_) as described above. Estimates were adjusted for age and racial/ethnic group when not stratified by race. Women were categorised into four levels of age (<64, 65–68, 69–72, 73+). All analyses were performed in SAS v8 (SAS Insitute, Cary, NC, USA).

## Results

### Mean IGF1 plasma concentrations by racial/ethnic group

Table 1

**Table 1:** Characteristics and mean plasma IGF1 level (ng ml^−1^) by racial/ethnic group

**Variable**	**African American**	**Japanese American**	**Non-Latino White**	**Latino American**	***P*^a^**
No. of subjects (%)	123 (30)	71 (18)	58 (14)	154 (38)	
Mean age (years)	70.2	68.2	67.9	64.9	<0.0001
Mean height (in)	64.1	60.2	64.0	62.4	<0.0001
Mean weight (lb)	166.5	122.6	151.7	158.8	<0.0001
Mean BMI (kg m^−2^)	28.5	23.9	26.1	28.7	<0.0001
					
*Mean plasma IGF1 (ng ml*^*−1*^)*b*					
Crude	145 (135, 154)	145 (132, 158)	145 (130, 159)	118 (109, 127)	<0.0001
Age adjusted^c^	146 (136, 156)	144 (131, 157)	145 (130, 159)	116 (107, 125)	<0.0001

a *P*-value derived from ANOVA and analysis of covariance models.

b The associated 95% confidence limits are given in parentheses.

c Adjusted for age as a categorical variable.

shows characteristics and mean plasma IGF1 levels for the 406 healthy postmenopausal women selected for this analysis by racial/ethnic group. In our sample, African-American women had the oldest mean age at blood draw (70.2 years), while Latino-American women had the youngest mean age (64.9 years). Body size characteristics (weight, height) also differed significantly across racial/ethnic groups in our sample. African-American and Latino-American women had higher mean body mass index (BMI) scores (28.5 and 28.7, respectively) than White (26.1) or Japanese-American women (23.9). Latino-Americans had significantly lower mean plasma IGF-1 levels (118 ng ml^−1^ (crude) and 116 ng ml^−1^ (age-adjusted)). African-American, Japanese-American and Non-Latino White women had plasma IGF1 levels that did not differ significantly from each other, with age-adjusted means of 146, 144 and 145 ng ml^−1^, respectively. Further adjustment for BMI in the model did not change these results.

### IGF1 (CA)*_n_* genotype and mean IGF1 plasma concentrations by racial/ethnic group

IGF1 (CA)_*n*_ genotype results for a sample (*N*=230) of the 406 control women described above are shown in Table 2

**Table 2:** *IGF1* (CA)*_n_* microsatellite genotype frequencies among healthy postmenopausal women tested for plasma IGF1 level by racial/ethnic group

	**African American**	**Japanese American**	**Non-Latino White**	**Latino American**
**Genotype^a^**	***N*** **(%)**	***N*** **(%)**	***N*** **(%)**	***N*** **(%)**
23/17	0 (0)	1 (2)	0 (0)	0 (0)
22/19	1 (1.5)	0 (0)	3 (6.4)	1 (1.5)
21/21	2 (3.0)	2 (4.0)	0 (0)	2 (2.9)
21/20	1 (1.5)	4 (8.0)	1 (2.1)	3 (4.4)
21/19	8 (12.3)	12 (24.0)	5 (10.6)	11 (16.2)
21/18	3 (4.6)	5 (10.0)	0 (0)	2 (2.9)
21/17	1 (1.5)	2 (4.0)	1 (2.1)	0 (0)
21/16	1 (1.5)	0 (0)	0 (0)	0 (0)
20/20	3 (4.6)	0 (0)	1 (2.1)	2 (2.9)
20/19	7 (10.8)	8 (16.0)	12 (25.5)	14 (20.6)
20/18	2 (3.1)	0 (0)	0 (0)	1 (1.5)
20/16	1 (1.5)	0 (0)	0 (0)	0 (0)
19/19	10 (15.4)	5 (10.0)	21 (44.7)	29 (42.7)
19/18	13 (20.0)	3 (6.0)	3 (6.4)	2 (2.9)
19/17	1 (1.5)	6 (12.0)	0 (0)	1 (1.5)
19/16	3 (4.6)	0 (0)	0 (0)	0 (0)
18/18	4 (6.2)	2 (4.0)	0 (0)	0 (0)
18/17	3 (4.6)	0 (0)	0 (0)	0 (0)
18/16	1 (1.5)	0 (0)	0 (0)	0 (0)
				
Non-19/non-19	22 (33.9)	16 (32.0)	3 (6.4)	10 (14.7)
Non-19/19	33 (50.8)	29 (58.0)	23 (48.9)	29 (42.7)
19/19	10 (15.4)	5 (10.0)	21 (44.7)	29 (42.7)

a Number of (CA) repeats for the two alleles.

. In Non-Latino White and Latino-American women, the (CA)_19_ homozygote (19/19) is the most common genotype (44.7 and 42.7%, respectively), but for African-American women, the 19/18 genotype is most common (20.0%) and in Japanese-Americans, 21/19 is the most common genotype (24.0%). *χ*^2^ test for differences in the distribution of genotypes across racial ethnic groups are significant for the three-level categorisation strategy presented here (19/19, 19/non-19, non-19/non-19) and for the categorisation used by Rosen *et al*, (1998) (19/19 *vs* non-19). There is no evidence for departure from Hardy–Weinberg equilibrium in any of the racial/ethnic groups. In addition, we found no consistent statistically significant association between age and genotype among controls, cases or all women combined, providing no evidence for confounding by age.

In accordance with the findings previously described on the association between serum IGF1 levels and the (CA)_19_ genotype (Rosen *et al*, 1998), we compared mean plasma IGF1 concentration across three (CA)_*n*_ microsatellite genotype categories (non-19/non-19, non-19/19 and 19/19) (Table 3

**Table 3:** Mean plasma IGF1 level (ng ml^−1^) by (CA)*_n_* microsatellite genotype and racial/ethnic group^a^

**Genotype**	**African American**	**Japanese-American**	**Non-Latino White**	**Latino American**
Crude				
Non-19/non-19	150 (121, 180)	144 (119, 169)	160 (104, 216)	149 (107, 192)
Non-19/19	166 (142, 190)	148 (130, 167)	142 (121, 162)	126 (101, 151)
19/19	150 (106, 194)	146 (101, 190)	146 (125, 167)	118 (93, 142)
				
Adjusted^b^				
Non-19/non-19	149 (118, 179)	144 (120, 168)	157 (97, 217)	148 (103, 193)
Non-19/19	168 (142, 194)	147 (129, 165)	141 (121, 162)	126 (100, 153)
19/19	143 (97, 189)	143 (98, 187)	146 (124, 167)	117 (90, 144)

a The associated 95% confidence limits are given in parentheses.

b Adjusted for age.

). We found that the Latino-American women had the highest frequency of the (CA)_19_ homozygote genotype and the lowest mean plasma IGF1 level among the four racial/ethnic groups. Among Latino-American women there was an inverse relation between the number of 19 alleles and mean plasma IGF1 in the crude and age-adjusted analyses. However, this pattern was not evident in any other ethnic group. Furthermore, none of the differences in mean plasma IGF1 level across genotype were statistically significant.

### IGF1 (CA)*_n_* genotype and breast cancer risk

Table 4

**Table 4:** Characteristics of subjects^a^ included in the *IGF1* genotype/breast cancer risk analysis by racial/ethnic group

	**African American**	**Japanese American**	**Non-Latino White**	**Latino-American**	***P*-homo^b^**
**Variable**	**(cases/controls)**	**(cases/controls)**	**(cases/controls)**	**(cases/controls)**	**(cases/controls)**
No. of subjects	81/91	76/94	82/92	81/96	
Mean age (years)	68.1/67.9	68.5/68.4	68.9/67.6	68.4/67.0	0.85/0.47
*P* (*t*-test)^c^	0.88	0.85	0.15	0.13	
Mean height (in)	64.4/64.4	60.9/60.6	64.0/63.7	61.8/62.2	<0.0001/<0.0001
*P* (*t*-test)^c^	0.82	0.47	0.48	0.19	
Mean weight (lbs)	171.9/166.9	125.6/122.3	150.9/147.4	150.0/151.9	<0.0001/<0.0001
*P* (*t*-test)^c^	0.36	0.30	0.45	0.69	
Mean BMI (kg m^−2^)	29.2/28.3	23.8/23.4	26.0/25.6	27.7/27.6	<0.0001/<0.0001
*P* (*t*-test)^c^	0.30	0.41	0.58	0.85	

a Of the 373 controls selected for this case–control data set, 134 overlapped with controls selected for the plasma IGF1 by racial/ethnic group analysis (Tables 1, 2 and 3).

b *P*-value for homogeneity across all four racial/ethnic groups: cases/controls.

c *P*-value for difference between means between cases *vs* controls.

shows characteristics of the 693 subjects in the case–control analysis of the *IGF1* (CA)_19_ microsatellite and breast cancer risk. Ages of participants at blood draw ranged from 49 through 81 years. Mean age at blood draw did not differ significantly across racial/ethnic groups or between cases and controls in each racial/ethnic stratum. The pattern of body size characteristics (height, weight and BMI) in these 693 women is similar to those described for the 230 control participants in the mean IGF1 plasma analysis described above. As shown in Table 4, African-American women had the highest BMI scores (29.2 for cases and 28.3 for controls), Non-Latino White and Latino-Americans were intermediate and Japanese Americans had the lowest BMI scores (23.8 for cases and 23.4 for controls). Although body size differed significantly across racial/ethnic groups, differences were not significantly different between cases and controls.

The distribution of genotypes for the (CA)_*n*_ dinucleotide repeat among controls in the case–control analysis (data not shown) is similar to the distribution shown for the controls in the plasma IGF1 analysis shown in Table 1. Of the controls in the case–control analysis, 134 were included as controls in the analysis of plasma IGF1 levels by racial/ethnic group. The distribution of genotypes by case–control status is presented in Table 5. Results are presented for all stages of breast cancer combined (excluding ductal carcinoma *in situ*, (DCIS); *N*=320). Stratification by stage of disease (localised or regional and metastatic disease combined) did not significantly alter the results from those presented here. The 19/19 genotype was not predictive of low breast cancer risk in our sample of postmenopausal African-American, Japanese-American or Latino-American women. In Non-Latino Whites, the adjusted odds ratio for breast cancer risk associated with the 19/19 homozygote as compared to the baseline non-19/non-19 genotype was 0.82, but this protective effect was not statistically significant (95% CI 0.30–2.24) (Table 5

**Table 5:** Risk of breast cancer associated with *IGF1* (CA)*_n_* microsatellite genotype by racial/ethnic group^a^

**Race**	**Genotype**	**CA (%)**	**CO (%)**	**OR 95%CL^b^**	**OR 95%CL^c^**	***P*-trend**
African American	Non-19/non-19	24 (30)	39 (43)	1.00	1.00	
	Non-19/19	46 (57)	39 (43)	1.92 (0.99, 3.72)	1.99 (1.00, 3.96)	
	19/19	11 (14)	13 (14)	1.38 (0.53, 3.56)	1.32 (0.50, 3.47)	0.25
						
Japanese American	Non-19/non-19	27 (36)	32 (34)	1.00	1.00	
	Non-19/19	35 (46)	51 (54)	0.81 (0.42, 1.59)	0.81 (0.41, 1.63)	
	19/19	14 (18)	11 (12)	1.51 (0.59, 3.87)	1.46 (0.56, 3.82)	0.65
						
Non-Latino White	Non-19/non-19	12 (15)	8 (9)	1.00	1.00	
	Non-19/19	26 (32)	47 (51)	0.37 (0.13, 1.02)	0.35 (0.13, 0.99)	
	19/19	44 (54)	37 (40)	0.79 (0.29, 2.15)	0.82 (0.30, 2.24)	0.59
						
Latino American	Non-19/non-19	7 (9)	16 (17)	1.00	1.00	
	Non-19/19	46 (57)	43 (45)	2.44 (0.92, 6.52)	2.27 (0.84, 6.16)	
	19/19	28 (35)	37 (39)	1.73 (0.63, 4.77)	1.76 (0.62, 4.97)	0.40
						
All races combined	Non-19/non-19	70 (22)	95 (26)	1.00	1.00	
	Non-19/19	153 (48)	180 (48)	1.15 (0.79, 1.68)	1.14 (0.78, 1.68)^d^	
	19/19	97 (30)	98 (26)	1.34 (0.89, 2.04)	1.40 (0.90, 2.20)^d^	0.13^d^

a Of the 373 controls selected for this case–control data set, 134 overlapped with controls selected for the plasma IGF1 by racial/ethnic group analysis (Tables 1, 2 and 3).

b Crude.

c Adjusted for age as a categorical variable.

d Additionally adjusted for race.

). When all races are combined, the 19 allele seemed to be associated with an increased risk for breast cancer. When dichotomised into non-19 *vs* any-19 categories the risk effect was 1.21 (95% CI 0.83–1.75).

## Discussion

We found that circulating IGF1 levels in postmenopausal women differed significantly between Latino-American women and three other racial/ethnic groups. In our sample, Latino-American women had low IGF1 levels. African-American, Japanese-American and Non-Latino White women had higher circulating IGF1 levels relative to the Latino-American women. When analysing recent breast cancer rates calculated for the MEC, Pike *et al* found the highest rates of breast cancer among African-Americans followed by Japanese-Americans, Non-Latino Whites and Latino-Americans (Pike *et al*, 2002). The observed relative rates (compared to Whites) of Japanese-American, US-born Latinos, African Americans and Non-US-born Latinos are 1.00, 0.86, 0.83, 0.77, respectively. These relative rates were adjusted for seven breast cancer risk factors including age at and type of menopause, age at menarche, age at first birth, number of children, weight, HRT use and physical activity (Pike *et al*, 2002). In our MEC sample plasma IGF1 levels were thus high among the three racial/ethnic groups with high breast cancer rates and lowest among the racial/ethnic group with the lowest breast cancer rates. In accordance with the suggestion from previous epidemiological (Bohlke *et al*, 1998; Hankinson *et al*, 1998; Byrne *et al*, 2000) and experimental (Furlanetto and DiCarlo, 1984; Westley and May, 1994; Kleinberg, 1998) studies, our results support further investigation of the role of IGF1 in breast cancer aetiology.

We were unable to identify the *IGF1* (CA)_19_ genotype as a relevant genetic marker for cumulative lifetime exposure of breast cells to IGF1. In our study population of postmenopausal women from four different racial/ethnic backgrounds, the *IGF1* (CA)_19_ genotype was not consistently correlated with circulating IGF1 levels and thus explained neither the interindividual nor the inter-racial variation in blood IGF1 concentrations. Our result was consistent with the absence of an association in a recent study of Caucasian men (Allen *et al*, 2002), but contradicted the finding by Rosen *et al*, (1998) on the association between the (CA)_19_ dinucleotide repeat in the *IGF1* gene and low serum IGF1 levels in Caucasian men and postmenopausal women. Our results suggested that the previous finding by Rosen *et al* may have been a chance finding given that the categorisation of microsatellite alleles was not based on functional evidence. Alternatively, the genotype/phenotype correlation observed by Rosen *et al* may have been specific for their study population because of a nearby functional genetic variant in linkage disequilibrium with the microsatellite repeat.

In this study, we stratified analyses by racial/ethnic group. After stratification by race and genotype, the numbers in some strata became quite small, a fact that detracted from statistical power. There was some potential for random misclassification in genotype determination that may have biased our outcome towards null. Our careful control and checking process was designed to minimise the probability of this occurrence (see Materials and Methods). Some confidence has been gained by the fact that the distribution of allele frequencies was consistent with Hardy–Weinberg.

Future work in this area should involve analysis of larger multiethnic samples, exploration for relevant determinants and markers of IGF1 levels in the blood, in order to resolve the issue of causal involvement of IGF1 in the aetiology of postmenopausal breast cancer.
